# Supplementary material for: Assessment of statistical methods from single cell, bulk RNA-seq, and metagenomics applied to microbiome data
Source: Genome Biol. 2020 Aug 3;21:191. doi: 10.1186/s13059-020-02104-1 (PMC7398076; doi:10.1186/s13059-020-02104-1)
Supplement: Supplementary file 3 — Additional file 3: Review history. [file 13059_2020_2104_MOESM3_ESM.docx]

Review History

**First round of review**

**Reviewer 1**

**Are you able to assess all statistics in the manuscript, including the appropriateness of statistical tests used?**

Yes, and I have assessed the statistics in my report.

**Comments to author:**

This is a well written and thought out paper. In comparison with other papers that compare statistical tests for differential abundance, I think that the authors looked at a very sensible set of criteria. I was especially glad to see the inclusion of concordance, which is typically overlooked. Stratifying data sets into high, medium, and low diversity is also very clever, and more useful to the average microbial ecologist who would typically analyze a specific data structure. The figures are also very visually appealing. The authors are to be commended on their inclusion of scripts to reproduce the analysis. This more than anything is a major advance over the other comparisons of differential abundance methods for microbiome DA.

I have a number of suggestions

- Averaging over 10 simulations does not seem sufficient to get an accurate measure of the false positive rate (type 1 error rate simulation). I know that there are many data sets, but I think 1000 simulations is necessary: a confidence interval for the true proportion near p=0.01 has width 2*sqrt(p*(1-p)/n) = 2*sqrt(0.01*0.99/1000) = 0.006, while with only 10 simulations it is 0.06! I therefore don't think that the Type 1 error rate results are very precise. This absolutely needs to be fixed, even if the amount of computation increases enormously.

- In a similar vein, I think "consistency" (see note below) should require 100 random repeats, not 10.

- More details are needed regarding the WGS results. It wasn't clear to me what abundance was being investigated. Was it taxonomic relative abundance? Are they all from metaphlan2 taxonomic profiles? Context for WGS results should be stated very explicitly in the main text.

- I don't think the parametric simulations add to the manuscript. The results are predictable and they don't lead much insight. I actually think they weaken the manuscript and that they should be cut.

- I obviously have a COI here (this is a signed review), but corncob is gaining popularity and I think folks would be interested to see how it performs. It's quite fast and I think could be added to the analysis without too much difficulty. One reason why I suggest its inclusions is that it provides a more parsimonious and interpretable model for high sparsity without relying on zero inflation parameters, which are (IMO) totally arbitrary. There are 4 tests for corncob (Wald, LRT, parametric bootstrap Wald, parametric bootstrap LRT); I think all should be investigated since similar variations were done for the other methods.

- To a statistician, consistency means asymptotic convergence in probability of the estimator to the estimand. I recommend choosing a different word.

Overall I congratulate the authors on an interesting and careful study that is superior in many ways to other papers that investigate the same question (all the ones I know of are cited, too).

Sincerely,

Amy Willis

U Washington

**Reviewer 2**

**Are you able to assess all statistics in the manuscript, including the appropriateness of statistical tests used?**

Yes, and I have assessed the statistics in my report.

**Comments to author:**

The authors propose to benchmark methods developed for RNA-seq and scRNA-seq for differential abundance analysis in microbiome data on numerous real and simulated datasets. The idea of benchmarking is appealing, and the main take-home message from the manuscript is that scRNA-seq methods do not perform well on microbiome data. This is expected, as microbiome data are fundamentally different from scRNA-seq data, and RNA-seq data. The proposed study mainly focuses on the sparsity aspect of the data, but my main concern is that they do not address two other key inherent characteristic of microbiome data: their multivariate and compositionality nature. As such, I have the strong feeling that such benchmarking is not useful for the microbiome community - the unintended consequence would be that microbiome researchers apply the recommended methods mentioned in the manuscript, i.e. DESeq2 and limma-voom, that are not fully suited for this context of analysis.

My argumentation is as follows:

In the recent years the field of microbiome data analysis has made important progress and considers compositionality as a crucial issue. Key citations were referred in the discussion (p 15) but the argument of the authors is that there is still not consensus on whether microbiome data should be analysed as compositional (which is true, but that does not mean it should be only vaguely brushed). The authors argue that the compositional methods they benchmarked did not outperform other non-compositional methods. However only one method has been benchmarked, Aldex2 and despite what the Aldex2 authors state, this method is not necessarily considered as state-of-the-art approach in the microbiome field. Other studies independent to the Aldex2 authors have shown that the method has mild performances (Thorsen et al 2016, ref 12 in manuscript) and is highly conservative (e.g. ref 12 and Morton et al. 2019, Nature Comm https://doi.org/10.1038/s41467-019-10656-5 to cite a few). Whilst there is still a lack of methods to account for compositional data in microbiome research, I believe that the authors' argumentation is not fully justified. As the authors also highlight, the assumption of a Dirichlet multinomial distribution (on which Aldex2 relies) may not be suited for these data. In light with this, I am not totally convinced that the simulation schemes are realistic of microbiome compositional data.

Methods applied directly on count microbiome data are statistically flawed (seurat, MAST, scde), as was highlighted by Aitchison (ref 36) while the poscount normalisation (geometric mean on positive count) is not suited for compositional data. Current pragmatic solution adopted in the microbiome field is to CLR transform the data prior to analysis. CLR is similar to a TMM normalisation, as highlighted by the authors (ref 34), but normalisation itself does not solve compositional issues, especially when compositions between samples are very different.

My second point of argumentation is that microbiome data are inherently multivariate. Thus, any univariate test does not reflect the true nature of these data. This point has been largely ignored in the field, but new methods are arising based on log-contrast models (Lu et al. 2018, https://doi.org/10.1111/biom.12956), compositional balances (Rivera-Pinto et al. 2018 10.1128/mSystems.00053-18). Microbiome researchers still apply univariate tests in their quest of (univariate) p-values, but we should encourage them instead to look for microbial signatures or balances and shift their own paradigm.

Hence, I worry that such review will encourage researchers to use methods that are not fully suited for microbiome data. I acknowledge that appropriate methods for microbiome data are still lacking, but it is not just a matter of applying methods developed in RNA-seq or single cell RNA-seq that will address these issues that are not transferable.

**Authors Response**

**Point-by-point responses to the reviewers’ comments:**

*We thank both reviewers for their constructive comments that helped us greatly improve our manuscript. We provide below a point-by-point response to their criticisms, but first we summarize the main changes made with respect to the first version of the manuscript.*

*We have added to the comparison three new methods: corncob (both with likelihood ratio test and Wald test), songbird, and mixMC. The latter two are compositional methods, allowing us to compare compositional approaches.*

*We have updated the title to more appropriately describe the wide range of methods we included in our benchmark. The new title is: “Assessment of statistical methods from single cell, bulk RNA-seq and metagenomics applied to microbiome data”*

*To avoid relying solely on parametric simulations to study the power of the methods, we performed an enrichment analysis, looking at the ranking of anaerobic and aerobic genera in the contrast of the subgingival plaque (a strict anaerobic environment) versus the supragingival plaque (directly exposed to open air). This analysis allows us to compare the methods’ ability to find significant differences in taxa expected to be differentially abundant in the two sites.*

*We have down-weighted the contribution of the parametric simulations on the overall conclusions, to avoid favoring methods based on the data-generating models used for simulating the data. Furthermore, we have increased the number of random samples for the type I error and concordance analyses.*

Point-by-point response

Reviewer #1: This is a well written and thought out paper. In comparison with other papers that compare statistical tests for differential abundance, I think that the authors looked at a very sensible set of criteria. I was especially glad to see the inclusion of concordance, which is typically overlooked. Stratifying data sets into high, medium, and low diversity is also very clever, and more useful to the average microbial ecologist who would typically analyze a specific data structure. The figures are also very visually appealing. The authors are to be commended on their inclusion of scripts to reproduce the analysis. This more than anything is a major advance over the other comparisons of differential abundance methods for microbiome DA.

*We thank the reviewer for her enthusiasm towards our paper and we are glad that she enjoyed our work. We appreciate the several constructive points that she raised and we have implemented the proposed changes.*

I have a number of suggestions

- Averaging over 10 simulations does not seem sufficient to get an accurate measure of the false positive rate (type 1 error rate simulation). I know that there are many data sets, but I think 1000 simulations is necessary: a confidence interval for the true proportion near p=0.01 has width 2*sqrt(p*(1-p)/n) = 2*sqrt(0.01*0.99/1000) = 0.006, while with only 10 simulations it is 0.06! I therefore don't think that the Type 1 error rate results are very precise. This absolutely needs to be fixed, even if the amount of computation increases enormously.

*We have repeated the type I error analyses with 1000 simulations and the updated Figure 3 now shows the average over 1000 simulations. The overall message did not change, but there are a few cases in which the new results more clearly distinguish the performance of the compared methods. We updated the text describing Figure 3 accordingly.*

- In a similar vein, I think "consistency" (see note below) should require 100 random repeats, not 10.

*We have repeated the “consistency” (now referred to as concordance, see below) analysis using 100 rather than 10 random splits.*

- More details are needed regarding the WGS results. It wasn't clear to me what abundance was being investigated. Was it taxonomic relative abundance? Are they all from metaphlan2 taxonomic profiles? Context for WGS results should be stated very explicitly in the main text.

*The preprocessing of the WMS data is described in the vignette of the curatedMetagenomicData Bioconductor package (Chapter 4.1). Briefly, absolute raw count data are estimated from MetaPhlAn2 relative count data multiplied by the number of reads for each sample. We have added more details on the WMS data in the main text.*

- I don't think the parametric simulations add to the manuscript. The results are predictable and they don't lead much insight. I actually think they weaken the manuscript and that they should be cut.

*We agree with the reviewer that the results of the simulations are predictable and do not add much to the paper. However, we believe that it may be of interest to other researchers to look at our extensive simulation results to explore how specific aspects of the data generating distribution may influence the performance of a given method. For this reason, we have moved the results of the simulations in the supplementary material (Additional file 2) and we only briefly summarize them in the main text.*

- I obviously have a COI here (this is a signed review), but corncob is gaining popularity and I think folks would be interested to see how it performs. It's quite fast and I think could be added to the analysis without too much difficulty. One reason why I suggest its inclusions is that it provides a more parsimonious and interpretable model for high sparsity without relying on zero inflation parameters, which are (IMO) totally arbitrary. There are 4 tests for corncob (Wald, LRT, parametric bootstrap Wald, parametric bootstrap LRT); I think all should be investigated since similar variations were done for the other methods.

*We have added corncob (in addition to songbird and mixMC, see response to reviewer 2) to our comparison. Following the reviewer’s suggestion we have investigated the performance of both the Wald and the LRT test. Due to computational constraints, we did not include the parametric bootstrap version of these tests in our benchmark. We note that the corncob paper suggests the use of bootstrap tests for small sample sizes (n < 10) while all our comparisons have a sample size of at least n=40. However, we used the bootstrapped versions in a small WMS 5vs5 samples comparison.*

- To a statistician, consistency means asymptotic convergence in probability of the estimator to the estimand. I recommend choosing a different word.

*We thank the reviewer for this observation. We have made the following terminology changes:*

*What was previously referred to as “consistency” is now referred to as “between-method concordance”*

*What was previously referred to as “replicability” is now referred to as “within-method concordance”*

Overall I congratulate the authors on an interesting and careful study that is superior in many ways to other papers that investigate the same question (all the ones I know of are cited, too).

*We thank the reviewer again for the positive and constructive review.*

Reviewer #2: The authors propose to benchmark methods developed for RNA-seq and scRNA-seq for differential abundance analysis in microbiome data on numerous real and simulated datasets. The idea of benchmarking is appealing, and the main take-home message from the manuscript is that scRNA-seq methods do not perform well on microbiome data. This is expected, as microbiome data are fundamentally different from scRNA-seq data, and RNA-seq data. The proposed study mainly focuses on the sparsity aspect of the data, but my main concern is that they do not address two other key inherent characteristic of microbiome data: their multivariate and compositionality nature. As such, I have the strong feeling that such benchmarking is not useful for the microbiome community - the unintended consequence would be that microbiome researchers apply the recommended methods mentioned in the manuscript, i.e. DESeq2 and limma-voom, that are not fully suited for this context of analysis.

*We thank the reviewer for raising two important points that we address in our revision: the compositional and multivariate nature of microbiome data. In this revision, we have added differential abundance methods developed specifically for microbiome data analysis, including two explicitly compositional methods, and have expanded our discussion of these issues. This will be especially relevant to the field because univariate approaches are in practice the dominant approach to inference (as opposed to prediction) across many types of high-dimensional omic data, including metagenomics. This benchmarking leverages both 16S and WMS datasets from the Human Microbiome Project in a context where there is a single relevant covariate and expected biological results: that oxygenated environments (such as supragingival plaque) favor aerobic bacteria, while non-oxygenated environments (such as subgingival plaque in the same participants) favor anaerobic bacteria. This analysis provides empirical evidence that univariate modeling should not yet be discounted for the identification of relevant microbiome features. To the best of our knowledge, this is the first benchmark to compare compositional and univariate differential abundance methods using real datasets with an expected biological result, going beyond simulations and demonstrative examples. We conclude, however, that the ideal method for microbiome data is not yet available, but that this benchmarking will help that effort by identifying the shortcomings of existing methods.*

My argumentation is as follows:

In recent years the field of microbiome data analysis has made important progress and considers compositionality as a crucial issue. Key citations were referred to in the discussion (p 15) but the argument of the authors is that there is still not consensus on whether microbiome data should be analysed as compositional (which is true, but that does not mean it should be only vaguely brushed).

The authors argue that the compositional methods they benchmarked did not outperform other non-compositional methods. However only one method has been benchmarked, Aldex2 and despite what the Aldex2 authors state, this method is not necessarily considered as state-of-the-art approach in the microbiome field. Other studies independent to the Aldex2 authors have shown that the method has mild performances (Thorsen et al 2016, ref 12 in manuscript) and is highly conservative (e.g. ref 12 and Morton et al. 2019, Nature Comm https://doi.org/10.1038/s41467-019-10656-5 to cite a few). Whilst there is still a lack of methods to account for compositional data in microbiome research, I believe that the authors' argumentation is not fully justified. As the authors also highlight, the assumption of a Dirichlet multinomial distribution (on which Aldex2 relies) may not be suited for these data.

*First, we want to emphasize that other data resulting from sequencing, most notably RNA-seq, are also inherently multivariate and compositional. As previously shown in RNA-seq data, count models provide a good approximation of compositional data when the number of features and the total number of sequenced reads are large.*

*However, as the reviewer points out, compositionality may have greater importance in some microbiome data due to the potential presence of dominant microbes. We agree that the manuscript lacked adequate consideration of compositional methods, and on the need to assess whether their benefits outweigh other drawbacks they may introduce, as seems to be the consensus in RNA-seq.*

*To this end we have included two recent methods, songbird [1] and mixMC [2], that, along with ALDEx2, provide a good representation of available compositionality-aware methods for microbiome analysis. Furthermore, we have added a paragraph in the Discussion to highlight the differences in the performance of compositional and non-compositional methods.*

In light with this, I am not totally convinced that the simulation schemes are realistic of microbiome compositional data.

*We realize that our simulation scheme may have skewed the comparison in favor of count models that assume the negative binomial or zinb distributions, and have downweighted its importance in the manuscript in favor of analysis of experimental data. Following a suggestion by reviewer 1, we have moved the simulation analysis to the supplementary material and given less emphasis to these results in the discussion and conclusions.*

*The main purpose of our simulation study was to compare the power of each method. To fill this gap in the revised version of our manuscript, we perform an enrichment analysis, looking at each method’s ranking of anaerobic and aerobic taxa in the contrast of the subgingival plaque (a strict anaerobic environment) versus the supragingival plaque (directly exposed to open air). This analysis allows us to compare the methods’ power without relying on parametric simulations.*

Methods applied directly on count microbiome data are statistically flawed (seurat, MAST, scde), as was highlighted by Aitchison (ref 36) while the poscount normalisation (geometric mean on positive count) is not suited for compositional data. Current pragmatic solution adopted in the microbiome field is to CLR transform the data prior to analysis. CLR is similar to a TMM normalisation, as highlighted by the authors (ref 34), but normalisation itself does not solve compositional issues, especially when compositions between samples are very different.

*While all available methods require simplifying assumptions that may not always hold up, we strongly disagree that applying methods directly on count data is fatally flawed. As discussed briefly in our discussion, count models are good approximations for compositional data when both the number of features and the total number of sequenced reads are large (cf. Poisson approximation of multinomial).*

*As the reviewer points out, CLR transformation is a pragmatic solution to address compositionality, and many normalizations proposed in the RNA-seq field can be used for the same purpose. We do agree with the reviewer that standard normalization is not sufficient when compositions between samples are very different. In fact, as any statistical model, normalization methods have assumptions, and when the assumptions are not met the methods can have poor performances. In the case of TMM and geometric mean normalization the assumptions are: (i) the majority of the taxa are not differentially abundant between the compared groups, (ii) the number of over-abundant taxa is roughly equivalent to the number of under-abundant taxa. It has been shown, in RNA-seq data, that only when both (i) and (ii) are not met, normalization can work poorly. In these cases, alternative approaches based on reference sets or controls may be preferable.*

*We have now included one method, songbird, that addresses compositionality in a different way, i.e., by using “reference frames”. Our concordance analysis highlights cases in which this method performs very differently than all the others, suggesting additional care in the interpretation of the results. Overall, our analyses empirically show a reasonable agreement between songbird and the other methods, suggesting that, even for our most extreme comparison (tongue dorsum vs. stool) normalization works well.*

My second point of argumentation is that microbiome data are inherently multivariate. Thus, any univariate test does not reflect the true nature of these data. This point has been largely ignored in the field, but new methods are arising based on log-contrast models (Lu et al. 2018, https://doi.org/10.1111/biom.12956), compositional balances (Rivera-Pinto et al. 2018 10.1128/mSystems.00053-18). Microbiome researchers still apply univariate tests in their quest of (univariate) p-values, but we should encourage them instead to look for microbial signatures or balances and shift their own paradigm.

*We agree with the reviewer that microbiome data (as any “omic” data) are inherently multivariate. Ideally, multivariate approaches have the advantage of modeling the complex dependencies among features; however, since the sample size is often 1-2 orders of magnitude smaller than the number of features (“large p, small n” problem), multivariate models may suffer from low power and computational instability.*

*We also agree with the reviewer that exploring multivariate approaches for microbiome data is a fascinating research question. However, these methods are still not mature enough to be included in our benchmark, either because of lack of a stable implementation, or for issues with scaling to more than 40-50 OTUs.*

*We have nonetheless included one multivariate approach, mixMC, in our benchmark and we discuss three interesting novel approaches (including the two suggested by the reviewer) in the conclusions section to provide an outlook of the field.*

Hence, I worry that such review will encourage researchers to use methods that are not fully suited for microbiome data. I acknowledge that appropriate methods for microbiome data are still lacking, but it is not just a matter of applying methods developed in RNA-seq or single cell RNA-seq that will address these issues that are not transferable.

*We would like to clarify that the intent of this benchmark was not to discourage research into novel methods for microbiome data. The intent of this benchmark is two-fold: we provide (i) a resource for the practitioner to choose the best method among the ones available to them, and (ii) a framework for developers of statistical methods to benchmark their novel method against the state-of-the-art approaches.*

*We fully realize that there is room for improvement and that developing methods to specifically address some of the unique characteristics of metagenomics data will likely contribute to improving our understanding of the microbiome. We have modified the abstract and the conclusions to reflect this.*

*On the other hand, we would like to emphasize that only by benchmarking existing methods can we understand what are the open questions that new methods must solve. Our work empirically shows that many of the issues addressed by RNA-seq or single-cell RNA-seq methods are indeed transferable to microbiome data and that novel bespoke methods may benefit from lessons learned in the transcriptomic field.*

*References:*

*1. Morton JT, Marotz C, Washburne A, Silverman J, Zaramela LS, Edlund A, et al. Establishing microbial composition measurement standards with reference frames. Nat Commun. 2019;10:2719.*

*2. Lê Cao K-A, Costello M-E, Lakis VA, Bartolo F, Chua X-Y, Brazeilles R, et al. MixMC: A Multivariate Statistical Framework to Gain Insight into Microbial Communities. Moreno-Hagelsieb G, editor. PLOS ONE. 2016;11:e0160169.*

**Second round of review**

**Reviewer 1**

The authors have addressed all of my concerns and I congratulate them on an excellent evaluation that is likely to be useful in guiding the field forward.

**Reviewer 2**

I am very satisfied with the revision of this manuscript, which present a comprehensive benchmarking and will be useful for the microbiome community. Well done.
